# Vincent Cristofalo “Rising Star”Award: DNA Methylation Landscapes in Aging

**DOI:** 10.1093/geroni/igab046.1492

**Published:** 2021-12-17

**Authors:** Morgan Levine

**Affiliations:** Yale University, New Haven, Connecticut, United States

## Abstract

The epigenetic code can be thought of as the operating system of the cell. It controls the most basic and critical cellular processes including differentiation, replication, metabolism, and signaling. Yet, with age, the epigenetic landscape is remodeled, bringing about widespread consequences for cellular and tissue identity, integrity, and functioning. But, what if like computer programmers, we could discover how to recode or restore the original program? The revolutionary discoveries by Yamanaka and Takahashi suggests this may be possible. While early experiments showed that Yamanaka factors could be used to convert somatic cells into induced pluripotent stem cells, more recent work by us and others have shown that signatures of epigenetic aging are also wiped clean during this process. What’s more, epigenetic age reversal appears to take place early in the process and thus can be achieved without the cell _needing to dedifferentiate. Building off of this discovery, our lab is combining novel experiments and advanced bioinformatic techniques to decipher the epigenetic code and determine how it is remodeled during aging, development, and reprogramming. In our recent work, we have made advancements in mapping the epigenetic alterations observed in aging and linking them to both cellular processes and disease etiology. We have identified specific age changes in mouse and human cells that reflect mitotic history, cellular senescence, oxidative damage, and mitochondrial dysfunction. We have also demonstrated that these changes inform differences in organismal lifespan and/or disease etiology at the tissue level. Overall, this work has sweeping implications for our basic understanding of epigenetic aging and reprogramming, and will help provide the foundation for potent therapeutics that extend healthspan and lifespan.

